# Benefits of Affective Pedagogical Agents in Multimedia Instruction

**DOI:** 10.3389/fpsyg.2021.797236

**Published:** 2022-02-04

**Authors:** Yanqing Wang, Xiaowei Feng, Jiangnan Guo, Shaoying Gong, Yanan Wu, Jing Wang

**Affiliations:** ^1^Key Laboratory of Adolescent Cyberpsychology and Behavior, Central China Normal University, Ministry of Education, Wuhan, China; ^2^School of Psychology, Central China Normal University, Wuhan, China; ^3^Student Affairs Office, Shijiazhuang University, Shijiazhuang, China

**Keywords:** affective pedagogical agents, multimedia learning, emotions, motivation, learning

## Abstract

The goal of the present study is to explore whether the affective states (happy or neutral) of a pedagogical agent (PA) in an online multimedia lesson yields different learning processes and outcomes, and whether the effects of affective PAs depend on the learners’ emotion regulation strategies and their prior knowledge. In three experiments, undergraduates were asked to view a narrated animation about synaptic transmission that included either a happy PA (smiling expression and enthusiastic voice) or a neutral PA (neutral expression and calm voice) and subsequently took emotions, motivation, cognitive outcomes tests. Across three experiments, the happy PA group reported more positive emotions (*ds* = 0.70, 0.46, and 0.60) and higher level of motivation (*ds* = 0.76, 0.49, and 0.51) than the neutral PA group. Moreover, the happy PA prompted higher germane load (*d* = 0.41) than a neutral PA in Experiment 3. However, adding a happy PA to the screen did not improve learning performance. In addition, in Experiment 2, learners’ usage of emotion regulation strategies moderated the effectiveness of affective PA on positive emotions in learners. Specifically, happy PAs increased the positive emotions of students who used expressive suppression strategy (*d* = 0.99) but not those who used cognitive reappraisal strategy (*d* = 0.13). In Experiment 3, the effectiveness of affective PAs was not moderated by learners’ prior knowledge. Results support the cognitive affective theory of learning with media (CATLM) that students are happier and more motivated when they learn from happy PAs than from neutral PAs.

## Introduction

### Objective and Framework

How to design video lectures to arouse learners’ positive emotions, and will such positive emotions affect learning? Prior research has mostly focused on the question of how to design learning materials to foster affective processing in multimedia instruction (Um et al., 2012; Plass et al., 2014; Shangguan et al., 2019). In recent years, advances in computer technology and intelligent tutoring systems have enabled instructional designers to embed an animated pedagogical agent in computer-based learning environments. The pedagogical agent (PA) is a character that is presented on a screen to deliver instruction through verbal and non-verbal communication (Moreno, 2005; Veletsianos and Russell, 2014; Lin et al., 2020; Treal et al., 2020). In this case, researchers are increasingly concerned on how to incorporate emotional design elements into PAs (e.g., affective pedagogical agents) to arouse learners’ positive emotions and motivation, thus improving learning. Affective pedagogical agent (affective PA) is a type of agent that is designed to elicit certain affective experiences in learners through multiple modalities such as facial expressions, voices, and gestures (Guo and Goh, 2015). The goal of the present study is to explore the effects of affective PAs in a multimedia narrated video, and further identify the important boundary conditions that impact affective PAs.

### Literature Review

#### Affective Pedagogical Agents in Multimedia Learning

During the past 10 years, research examining the influence of emotional design on multimedia learning has proliferated. Emotional design refers to the way of redesigning learning environments with the goal to increase learners’ positive emotions and motivation to enhance learning performance (Um et al., 2012; Mayer and Estrella, 2014; Beege et al., 2020; Cheng et al., 2020; Wang X. et al., 2020). Emotional design includes two ways (Plass and Schwartz, 2014; Plass and Kaplan, 2016): One involves the emotional design of online learning materials and the other is the emotional design of interactive features in multimedia learning environments (e.g., the emotional stances of PAs).

Applying emotional design principles to learning materials pioneered first by Um et al. (2012). In their study, undergraduates were asked to learn a computer-based lesson covering the topic “how immunization works.” In the positive emotional design (PED) lesson, the essential elements were rendered with warm colors, round shapes, and anthropomorphic eyes, while the control lesson was designed in monochromatic grayscale and rectangular shapes. The results found that college students in the PED group reported more positive emotions, lower task difficulty, higher level of motivation and performed better on comprehension and transfer tests than those in the neutral emotional design (NED) group. Subsequently, a growing number of studies found that PED could prime positive emotional response in learners, which in turn resulted in better learning outcomes (Plass et al., 2014; Gong et al., 2017; Uzun and Yıldırım, 2018; Shangguan et al., 2020). A recent meta-analysis by Wong and Adesope (2021) corroborated the findings from these studies, showing the positive effects of emotional design on learning outcomes (*g*_*retention*_ = 0.35; *g*_*transfer*_ = 0.27; *g*_*comprehension*_ = 0.29). Builds on our understanding of PED in learning materials, the present study investigates the emotional design of PAs (affective PAs), which fits within the second way of emotional design.

There was also preliminary evidence showing that a positive affective PA including enthusiastic voices, smiling facial expressions and happy gestures could induce positive emotions in learners, improve motivation (Baylor and Kim, 2009; Liew et al., 2017; Wang et al., 2019; dos Santos Alencar and de Magalhães Netto, 2020; Schneider et al., 2022) and learning performance (Hernández et al., 2009; Beege et al., 2020). For example, Liew et al. (2017) applied the emotional design principle in an interactive learning environment by designing an enthusiastic PA to constantly smile, nod, and provide enthusiastic remarks. The results found that college students in the enthusiastic agent condition reported more positive emotions, higher intrinsic motivation and performed better on learning outcomes than learners in the neutral agent condition. Wang et al. (2019) asked college students to watch three different video lectures: the heightened level of expressiveness lecture (e.g., expressive facial expression), the conventional level of expressiveness lecture (e.g., neutral facial expression) and the audio-only lecture (no instructor’ image). On subsequent tests, students in the video lectures with a heightened level of expressiveness instructor reported higher arousal level and learning satisfaction and scored higher in the medium-term recall test. The results again indicated the power of affective PAs on affective processing and cognitive outcomes. Similarly, Schneider et al. (2022) found that a PA who performed facial expressions led to higher perception of learning facilitation and better transfer performance compared with a PA who without facial expressions. Guo and Goh (2015) conducted a meta-analysis involving 30 experiments and found that the use of affective PAs had a moderate effect size of motivation (*r* = 0.35) and relatively smaller impacts on retention (*r* = 0.29) and comprehension (*r* = 0.26).

Two theories were used to explain the effectiveness of affective PAs in multimedia learning environments. The first is emotional response theory (Russell and Mehrabian, 1974), which emphasizes the relationship between students’ perceptions of teacher immediacy behaviors and their emotional responses and cognitive learning. Based on this theory, Mottet et al. (2006) further explicated three components in instructional contexts: (1) instructors’ verbal and non-verbal communications; (2) learners’ emotional responses (3) learners’ approach-avoidance behaviors. When the verbal and non-verbal messages of a PA increased positive emotions in learners, they would occur approach behaviors in terms of learning (Horan et al., 2012). From the perspective of emotional response theory, PAs with enthusiastic voices, smiling faces, and expressive gestures could elicit positive emotional responses in learners and promote them to engage in learning-related activities (Liew et al., 2017).

The second is the Cognitive Affective Theory of Learning with Media (CATLM, Moreno and Mayer, 2007), which extended the Cognitive Theory of Multimedia Learning (CTML; Mayer, 2021) by adding motivational and affective factors. CATLM proposes three assumptions: First, affective mediation hypothesis holds that motivation and affective factors may mediate learning by increasing or decreasing cognitive engagement; Second, metacognitive mediation hypothesis refers to individual meta-cognitive skills may influence learning by affecting cognitive and emotional processes; Third, individual differences hypothesis argues that individual characteristics may moderate the effectiveness of multimedia learning. According to the affective mediation hypothesis of CATLM, when PAs display positive emotions during online learning, learners may experience four key steps (Horovitz and Mayer, 2021; Lawson et al., 2021): (1) the learners first need to recognize the PA’s positive emotions; (2) the learners respond to the PA’s affective stances (such as feeling the same emotions as the affective PAs); (3) the learners’ positive emotions improve the level of motivation to engage in deep cognitive processing; (4) the motivational states lead to better learning outcomes.

Some studies have found the positive effects of affective PAs on arousing learners’ positive emotions and motivation, but positive affective processing did not necessarily facilitate learning performance (Guo et al., 2014, 2015; Guo and Goh, 2016; Horovitz and Mayer, 2021). For instance, Horovitz and Mayer (2021) asked college students to watch an instructional video on the statistical topic of binomial probability, students in the happy instructor group could recognize the emotional state of the instructor and rated themselves as happier and more motivated than those in the bored instructor group. However, there were no significant differences in learning outcomes among different types of instructors. A series of studies by Guo et al. (2014, 2015) asked university students to interact with affective embodied agents that expressed positive affective through facial expression, body gesture and scripted feedback or neutral embodied agents. They found that affective embodied agents group reported more enjoyment and higher level of motivation than neutral embodied agents, but there was no difference in learning outcomes between the two groups.

In contrast to these findings, some studies demonstrated that affective PAs neither induced learners’ positive emotions nor enhanced learning (Beege et al., 2020; Xie, 2020, Experiment 1). There are even research findings showed that the positive facial expression (e.g., smile) of PAs led to negative emotional and motivational responses in learners (Liew et al., 2016), or resulted in poorer comprehension test performance (Frechette and Moreno, 2010). The mixed findings indicated that additional factors may constrain the effectiveness of affective PA. Although there are some researchers tried to address the debates in the literature by identifying potential moderating variables, such as the types of affective PAs (Horovitz and Mayer, 2021) and the channel of emotional cues (Ba et al., 2021). They have still ignored the importance of learners’ individual characteristics (e.g., learners’ emotion regulation strategies and prior knowledge). According to the individual differences assumption of the CATML, individual characteristics may affect the efficacy of instructional design in multimedia learning. Therefore, it is necessary to examine whether learners’ individual characteristics were important boundary conditions for the effectiveness of affective PAs.

#### Learners’ Emotions Regulation Strategies

Emotion regulation is the set of controlled and automatic processes that individuals exert influence on how they experience or express their emotions and attempt to regulate or change the trajectory, duration, and intensity of emotions (Webb et al., 2012; Gross, 2015). The process model of emotion regulation (Gross, 1998) points out five emotion regulation strategies: situation selection, situation modification, attentional deployment, cognitive reappraisal, and expressive suppression. Cognitive reappraisal and expressive suppression are the two most commonly used emotion regulation strategies. The former is a form of cognitive change that refers to altering the emotional state by reformulating the meaning of a situation from other perspectives and reinterpreting the situational stimulus (Gross and Thompson, 2007). The latter is a form of response modulation that refers to the deliberate suppression of an impending or ongoing emotional expression, such as “putting a smile on” when angry. Studies have found that students using cognitive reappraisal strategy may be more confident in regulating their emotional experience, and thereby expressing more positive emotions but less negative emotions (Goldin et al., 2008). Compared to the cognitive reappraisal strategy, the expressive suppression strategy is mainly used to regulate the external emotional response rather than the internal emotional state. Therefore, learners who used expressive suppression strategy are more likely to experience more negative emotions and less positive emotions (Dryman and Heimberg, 2018). Similarly, research has shown that the cognitive consequences of different emotion regulation strategies may be different. For example, Strain and D’Mello (2015) found that students who used cognitive reappraisal strategy reported more affective engagement and achieved better learning outcomes than those who did not use any strategy. Dillon et al. (2007) reported that cognitive reappraisal strategy (cognitive up-regulation and cognitive down-regulation) rather than expressive suppression strategy promoted the memory of emotional materials. According to this line of research, learners who used cognitive reappraisal strategy can successfully regulate their emotional experience during learning. By contrast, the expressive suppression strategy is considered as a maladaptive emotion regulation strategy, which is usually associated with negative emotional experience and cognitive consequences, so students who used expressive suppression strategy may need more affective aid which provided by affective PAs. Therefore, we predict that affective PAs may be more beneficial to learners who used expressive suppression strategy than learners who used cognitive reappraisal strategy.

#### Learners’ Prior Knowledge

Learners’ prior knowledge refers to the level of their experience in a particular domain, which is regarded as one of the most important individual characteristics that affect learning (Kalyuga et al., 2003). Prior research has found that the level of prior knowledge may affect students’ cognitive processing and learning outcomes (Kalyuga, 2007). A schema-based approach can be used to explain the differences between experienced and inexperienced learners. According to the experience dominance effect, learners with high prior knowledge possess a large number of relevant knowledge schemas stored in long-term memory. When new information is presented to learners, high-knowledge learners can quickly connect the input knowledge with existing schemas and avoid processing overwhelming amounts of information at once. By contrast, learners with low prior knowledge may lack sophisticated schemas associated with learning materials and have difficulty in processing relevant information in a timely manner, thus reducing the cognitive resources for organization and integration. In terms of learning performance, high-knowledge learners leave more available cognitive resources to process the central concepts, so perform better than low-knowledge learners. According to the expertise reversal effect (Kalyuga et al., 2003), instructional techniques that are effective for learners with low knowledge experience may be ineffective or even had negative consequences on those with high knowledge experience. When presented with a new learning material, low-knowledge learners are more likely to experience higher task difficulty due to lack of relevant schemas to guide cognitive processing, which may increase their negative emotions and decrease learning motivation (Efklides and Petkaki, 2005). Therefore, affective PAs may work as instructional supports to increase positive emotions and intrinsic motivation that stimulate and maintain generative processing. Instead, knowledgeable learners can apply schemas to knowledge construction on their own, so they may not need any instructional guidance (Shangguan et al., 2020; Wang F. et al., 2020). Therefore, the present study aims to explore the prediction that affective PAs may be more helpful for low prior knowledge learners than for high prior knowledge learners.

### The Present Study

Previous studies have found the effectiveness of affective PAs with single emotional cue (smiling expression or enthusiastic voice) (Liew et al., 2016; Beege et al., 2020) or multilevel emotional cues (smiling expressions, enthusiastic voices, high level of head movements and gestures, and additional remarks) (Liew et al., 2017; Horovitz and Mayer, 2021). However, among various emotional cues, facial expression and vocal expression were the essential attributes that influenced learners’ perceptions of the positive affection of agents (Liew et al., 2017). Domagk (2010) points out that the image and voice of PAs were the main factors priming the social interaction between learners and PAs. In light of this previous research, the affective PA in this study is designed with dual-channel emotional cues, including smiling facial expression and enthusiastic vocal expression. Thus, the first experiment of this study is conducted to explore whether affective PAs can affect learners’ emotions, motivation, cognitive processing, and learning outcomes. According to emotional response theory and CATLM, the presentation of an affective PA can help arouse positive emotions and improve learning motivation, causing the learners to exert more effort to engage in deep cognitive processing, which is more likely to lead to meaningful learning outcomes. Based on the emotional response theory and CATLM, we predict that affective PAs with smile expressions and enthusiastic voices can enhance learning outcomes (retention test and transfer test). Additionally, those students will report more positive emotions and higher level of intrinsic motivation (hypothesis 1).

Furthermore, the present study seeks to investigate whether the effectiveness of affective PAs is moderated by some potential factors such as learners’ emotion regulation strategies (Experiment 2) and prior knowledge (Experiment 3). According to the individual difference assumption of the CATLM, individual characteristics may affect the efficacy of instructional design in multimedia learning (Moreno, 2006). Based on the individual difference assumption of the CATLM and prior empirical studies, we hypothesize that compared to learners who used cognitive reappraisal strategy, learners who used expressive suppression strategy will report more positive emotions, higher level of intrinsic motivation, and achieve better learning outcomes when they receive an affective PA in contrast to a neutral PA (hypothesis 2). Besides, compared to learners with high prior knowledge, learners with low prior knowledge will report more positive emotions, higher level of intrinsic motivation, and achieve better learning outcomes when they receive an affective PA in contrast to a neutral PA (hypothesis 3).

## Experiment 1

### Method

#### Participants and Design

*A priori* power analysis was conducted using G*Power 3.1 with an estimated medium effect size *d* = 0.62, α = 0.05, power = 0.8 (Faul et al., 2007). The medium effect size was based on a prior study by Liew et al. (2017). Based on the analysis, the suggested total sample size was 66. Therefore, 70 undergraduates from Central China Normal University were recruited to take part in this experiment. Four participants were excluded because they did not complete the posttests. The final sample consisted of 66 participants. The mean age of them was 19.8 (*SD* = 1.23) and 52 of them were women. In a one-factorial between subjects-design, 33 participants served in the affective PA group and 33 in the neutral PA group. There were no significant differences among the groups on prior knowledge, *t*(64) = 0.90, *p* > 0.05, positive emotions, *t*(64) = 0.07, *p* > 0.05, mean age, *t*(64) = 0.20, *p* > 0.05, and proportion of men and women, χ^2^(1) = 0, *p* > 0.05.

#### Learning Materials

**FIGURE 1:**
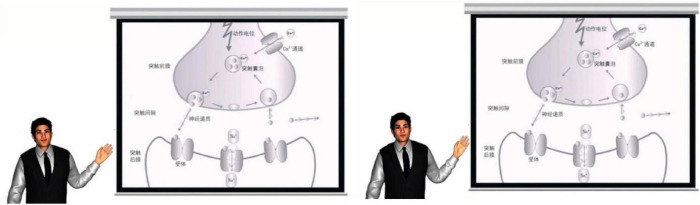
Example frames from multimedia learning materials: Affective pedagogical agent (Left) and neutral pedagogical agent (Right).

The materials were composed of two versions of computer-based instructional videos about the important process of synaptic transmission. The lesson focused on explaining how the chemical signals were transmitted across neurons in the nervous system, and the functions of action potentials, calcium ions, synaptic vesicles, and neurotransmitters in the transmission process. The same learning materials have been used in previous studies (Wang et al., 2018; Li et al., 2019; Wang F. et al., 2020), which proved to be moderately difficult. Both versions consisted of oral narration in a man voice and an illustration depicting the parts of neurons that are involved in synaptic transmission (as exemplified in Figure 1). For the affective PA condition, there was a middle-aged agent standing next to the illustration who displayed happy facial expressions and enthusiastic voices. In line with Liew et al. (2017, 2020), the enthusiastic agent was designed to constantly smile and the emotional tone of the voice was enthusiastic (i.e., a large dynamic pitch variation and a high pitch contour were used). In contrast, the neutral PA used neutral facial expressions and serious and calm voices (i.e., a low pitch level and small pitch variations were used). A professional male voice actor recorded the speech for the enthusiastic and neutral agent. The videos were created by Flash CS6 with the screen size is 1680 × 1050 pixels. Each video lasted 128 s.

#### Assessment Instruments

##### Pretest

The pretest consisted of a demographic survey (such as age, gender, educational level, and major), a knowledge questionnaire, and an emotional state scale. All materials were in Chinese.

The knowledge questionnaire was used to assess the level of prior knowledge concerning the chemical synaptic transmission, including 10 multiple-choice questions (e.g., “When the cell is in a resting state, what are the characteristics of the electric potential inside and outside the cell membrane?”) and four self-evaluated questions (e.g., “How much do you know about chemical synapses?” “Have you taken any courses related to biological or neurophysiology”). There were four answers to each question on the multiple-choice questions and only one correct answer. Two points were awarded for each correct response. In terms of the subjective rating statements, participants were asked to mark a five-point scale ranging from 0 (very little) to 4 (very much) or a two-point scale marking 0 (No) or 2 (Yes). The total score of prior knowledge was computed by adding the number of points from all items, yielding the maximum points was 31. Similar prior knowledge questionnaires have been used in previous research (Wang et al., 2018; Wang F. et al., 2020).

The Positive Affective Scale (PAS) from the Positive and Negative Affect Schedule (PANAS; Watson et al., 1988) was used to assess students’ emotional baseline before formal learning. The PAS included 10 items: Enthusiastic, interested, determined, excited, inspired, alert, active, strong, proud, attentive, which were used to measure different feelings that learners experience in relation to positive affect. Participants were asked to rate emotions on a five-point Likert scale from 1 (not at all) to 5 (very much) before and after learning (coefficient α = 0.83 for PAS1, 0.9 for PAS2).

##### Posttest

The posttest included the same emotional state scale as the pretest, a motivation questionnaire, a cognitive load questionnaire and learning outcome tests (a retention test and a transfer test). To measure learners’ *intrinsic motivation*, participants completed a seven-point Likert-type Motivation Self-report Questionnaire developed by Isen and Reeve (2006). This questionnaire contains eight items, an example of the items was “The study materials aroused my desire to learn more.” Each item was rated from 1 (completely disagree) to 7 (completely agree). The total motivation score was computed by averaging the scores of the seven responds (α = 0.93).

*Cognitive load* experienced by learners was measured using the revised Cognitive Load Scale (Xiong et al., 2018). The scale consisted of 13 items, including three cognitive load subscales: internal cognitive load (ICL) (four items, Cronbach’s α = 0.85), external cognitive load (ECL) (four items, α = 0.81) and germane cognitive load (GCL) (five items, α = 0.83). Examples of the three subscales were “The explanation and description during the learning was very unclear” “The topics covered in the learning materials were very complex.” “The activity really enhanced my knowledge and understanding of synaptic transmission.” Each item was rated on a 10-point scale from 1 (completely disagree) to 10 (completely agree). Each individual’s score on cognitive load was computed by averaging their responses on each of subscales.

*Learning performance* was assessed using two learning outcome tests: retention test and transfer test. The retention test was comprised of seven fill-in-the-blank questions measuring the learners’ memorizing of key information of the instructional video. For example, “The chemical synaptic transmission between neurons is mainly carried out among _____, _____ and _____.” Participants received one point for each of information units (blanks), with a maximum of 22 points. The transfer test consisted of four open questions which required students to apply the newly learned knowledge to solve novel problems (e.g., “Cobra venom is rich in neurotoxins, so what do you think is the poisoning mechanism of being bitten by a cobra?”). One point was assigned for each acceptable statement regardless of wording, resulting in a total of 14 points. The measures used in this study are similar to those used in the previous studies by Wang F. et al. (2020). The test score was completed by two independent raters, and the average score of them was used as the learner’s final score. Inter-rater reliability on the retention test and the transfer test were *r* = 0.99 (*p* < 0.001) and *r* = 0.96 (*p* < 0.001), respectively.

#### Apparatus

The videos were presented on Dell PC computers with 24-inch monitors, and all participants wore headphones while watching the video.

#### Procedure

The participants were randomly assigned to the affective PA group or the neutral PA group and tested individually. First, participants read and filled in the informed consent form. Next, they were asked to complete the demographic questionnaire, the prior knowledge test, and the emotional subjective report questionnaire. Then, participants were informed that they would view a lesson about synaptic transmission, and they needed to complete the corresponding tests after learning. After watching the video, participants worked on the emotions and motivation questionnaires and cognitive outcomes tests. The total duration of the experiment was approximately 30 min. This study was approved by the ethics committee of the university.

### Results

Table 1 shows the mean scores and standard deviations on all variables for the affective PA group and the neutral PA group. We applied partial η^2^ or Cohen’s *d* as the effect size index. For the partial η^2^, the value of 0.01, 0.06, and 0.14 were considered as small, medium, and large effect sizes; For the Cohen’s *d*, the value of 0.20, 0.50, and 0.80 were considered as small, medium, and large effect size (Cohen, 1988), respectively.

**TABLE 1:** Means and standard deviations of all tests for two groups in Experiment 1.

Dependent variables	Affective PA		Neutral PA	
	*M*	*SD*	*M*	*SD*
Prior knowledge	14.61	5.4	15.88	6.11
The first positive emotions	3.39	0.53	3.38	0.53
The second positive emotions	3.63	0.56	2.98	0.33
Learning motivation	5.02	0.61	4.23	1.33
ICL	5.09	2.36	4.66	1.99
ECL	3.29	1.84	2.71	1.41
GCL	7.29	1.27	7.25	1.68
Retention test	14.17	4.82	13.64	4.60
Transfer test	3.01	1.64	3.11	1.42

*Affective PA, affective pedagogical agent; Neutral PA, neutral pedagogical agent; ICL, intrinsic cognitive load; ECL, external cognitive load; GCL, germane cognitive load.*

#### Were Affective Pedagogical Agents Effective in Arousing Learners’ Positive Emotions?

**FIGURE 2:**
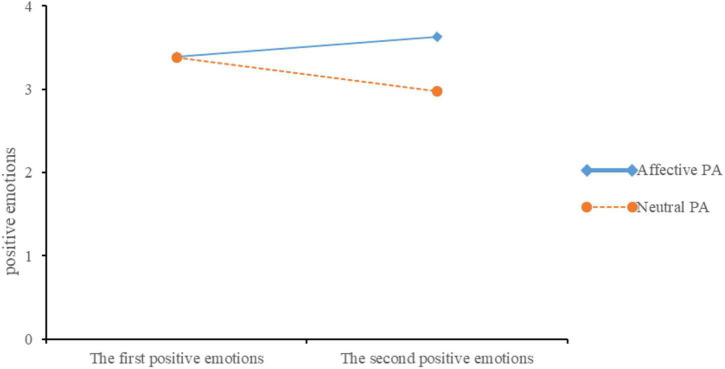
The positive emotions on the first and second measurement point for the two groups in Experiment 1.

To check whether adding an affective PA in multimedia courses can arouse learners’ positive emotions. we conducted a repeated measures analysis of variance (RM-ANCOVA) with the two measurement points of positive emotions as repeated measurement variables, the affective PA (affective PA and neutral PA) as between-subjects factor and prior knowledge score as a covariate. The analysis revealed a significant main effect for the affective PA, *F*(1,63) = 3.89, *p* = 0.05, $\eta_{\text{p}}^{2}$ = 0.058. The affective PA group (*M* = 3.51, *SD* = 0.45) reported more positive emotions than the neutral PA group (*M* = 3.28, *SD* = 0.54), and an interaction between the two measurement points of positive emotions and affective PA, *F*(1,63) = 7.67, *p* = 0.007, $\eta_{\text{p}}^{2}$ = 0.109. The simple effects analysis suggested that students reported more positive emotions at the second positive emotions measurement than the first positive emotions measurement in the affective PA group, *F*(1,63) = 4.11, *p* = 0.047, *d* = 0.44 (see Figure 2), but not in the neutral PA group, *F*(1,63) = 3.61, *p* > 0.05. However, there was no main effect for the measurement points of the positive emotions, *F* < 1.

#### Were Affective Pedagogical Agents Effective in Improving Learners’ Intrinsic Motivation?

Next, in order to investigate whether affective PA could affect learners’ intrinsic motivation, we performed a one-way ANCOVA with the two treatment groups as the between-subject factor and prior knowledge score as a covariate. As expected, analyses of the experimental result indicated that learners in the affective PA group reported higher intrinsic motivation than those in the neutral PA group, *F*(1,63) = 11.06, *p* = 0.002, *d* = 0.58.

#### Were Affective Pedagogical Agents Effective in Facilitating Cognitive Processing and Learning Outcomes?

To determine the effects of affective PA on cognitive processing and learning outcomes, we conducted one-way ANCOVAs using prior knowledge score as the covariate to compare the two groups on cognitive load, retention test, and transfer test.

With regard to the cognitive load, there was no significant difference between the affective PA group and the neutral PA group on ECL, *F* < 1, ICL, *F*(1,63) = 1.49, *p* > 0.05, and GCL, *F* < 1.

With regard to the learning outcomes, no statistically significant difference was found between the affective PA group and the neutral PA group on the retention test, *F*(1,63) = 1.79, *p* > 0.05, and transfer test, *F* < 1.

### Discussion

The goal of Experiment 1 was to investigate whether affective PAs with smiling facial expressions and enthusiastic voices could arouse learners’ positive emotions, increase intrinsic motivation and enhance learning performance from a narrated animation explaining the process of synaptic transmission. These results suggest that the affective PAs with smiling facial expressions and enthusiastic voices were effective in arousing the learners’ positive emotions and improving intrinsic motivation, which partly supports hypothesis 1. According to the emotional response theory, instructor behavior (communications) may affect learners’ emotional responses. Similarly, CATLM theory points that students are able to recognize the emotional states of the PAs, and feel the same emotions as the PAs, thereby triggering higher intrinsic motivation (Moreno and Mayer, 2007). Therefore, instructors’ positive affective states (e.g., verbal and non-verbal emotional cues) could elicit the same kind of emotions among students, and positive emotions in turn led to positive changes in learning motivation.

However, we found no support for the effects of affective PAs on retention and transfer performances. The finding was consistent with several previous studies, which reported that presenting an affective PA on the computer screen didn’t improve learning outcomes (Guo and Goh, 2016; Horovitz and Mayer, 2021). One possible reason may be that the learning outcomes tests utilized in the current study were immediate tests after learning. The effects of affective PAs on learning performance may be discerned on delay tests (Roediger and Karpicke, 2006). Another possibility may be that the duration of learning materials in our study was short and the influence of positive emotions on cognitive outcomes may be found in longer learning materials (Endres et al., 2020). It is of note that although the affective PAs did not increase the learners’ GCL that is necessary for making sense of the learning material, the novelty of affective PAs did not cause learners to engage in more external cognitive processing. The result indicated that affective PAs did not serve as irrelevant information to impede learning.

## Experiment 2

Experiment 1 found the positive effects of adding an affective PA on learners’ positive emotions and intrinsic motivation. The goal of Experiment 2 is to test whether the positive effects of affective PAs depend on the emotion regulation strategies learners used, that is whether affective PAs are more beneficial for learners who used expressive suppression strategy than for those who used cognitive reappraisal strategy.

### Method

#### Participants and Design

A total of 482 participants enrolled at a university in central China were recruited to complete an Emotion Regulation Questionnaire (ERQ). Then, according to their scores on cognitive reappraisal dimension and expressive suppression dimension, the top 27% of the students in two strategies were, respectively, classified as cognitive reappraisal (CR) strategy group or expressive suppression (ES) strategy group. We eliminated data from students who studied biology (65), data from students who did not complete the questionnaire (9), and data from students who did not respond to the majority of survey questions (8). The final sample consisted of 111 participants. Among them, 59 used CR strategy and 52 used ES strategy. *A priori* power analysis was conducted using G*Power 3.1 with a medium effect size of *f* = 0.30, α = 0.05, power = 0.8 (Faul et al., 2007). Based on the analysis, the suggested total sample size was 102. Paired sample *t*-tests showed that there was a significant difference between the two groups in CR strategy, *t_cognitive reappraisal_* (109) = 17.06, *p* < 0.001, and ES strategy, *t_expressive suppression_* (109) = 14.09, *p* < 0.001. The average age of the participants was 19.51 years (*SD* = 0.94), and 93 of them were women.

Participants were randomly assigned to one of the four conditions that resulted from a 2 × 2 between-factors design with affective PA (affective PA vs. neutral PA) and emotion regulation strategies (CR strategy vs. ES strategy) as factors. There were 28 in the affective PA/CR strategy group; 31 in the affective PA/ES strategy group; 25 in the neutral PA/CR strategy group; 27 in the neutral PA/ES strategy group. There were no significant differences among four groups on prior knowledge, *F*(3,107) = 1.68, *p* > 0.05, positive emotions, *F* < 1, and proportion of men and women, χ^2^(3) = 5.66, *p* > 0.05.

#### Materials and Apparatus

The learning materials, pretest (prior knowledge test and emotional state scale) and posttest (motivation questionnaire, cognitive load scale, retention test and transfer test) were the same as in Experiment 1. Inter-rater reliability was *r* = 0.99 (*p* < 0.001) for the retention test and *r* = 0.97 (*p* < 0.001) for the transfer test.

The Chinese version of ERQ revised by Wang et al. (2007) was used to measure learners’ usage of two emotion regulation strategies: cognitive reappraisal and expressive suppression. It was originally developed by Gross and John (2003) and had since been translated into 33 languages. Separate scale scores were derived for these two emotion regulation strategies. The cognitive reappraisal scale consists of six items (α = 0.87), for example, “When I want to feel more positive emotions (e.g., enjoyment), I change what I’m thinking about.” The expressive suppression scale consists of four items (α = 0.60), for example, “I control my emotions by not expressing them.” All items use a seven-level rating scale ranging from 1 (completely disagree) to 7 (completely agree). The apparatus was the same as in Experiment 1.

#### Procedure

The procedure was the same as in Experiment 1. In addition, participants who commonly used each regulation strategy were randomly assigned to two conditions.

### Results

Table 2 presents the means and standard deviations of the four groups on all variables. To explore the effects of affective PA and emotion regulation strategies on learners’ positive emotions, intrinsic motivation, cognitive load and learning outcomes, we conducted a two-way ANCOVA with affective PA (affective PA vs. neutral PA) and emotion regulation strategies (CR strategy vs. ES strategy) as factors, and prior knowledge score as the covariate.

**TABLE 2:** Means and standard deviations of all tests for four groups in Experiment 2.

Dependent variables	Affective PA				Neutral PA			
	CR strategy		ES strategy		CR strategy		ES strategy	
	*M*	*SD*	*M*	*SD*	*M*	*SD*	*M*	*SD*
Prior knowledge	16.18	5.81	14.6	5.94	12.52	6.66	14.22	6.65
The first positive emotions	3.54	0.64	3.46	0.42	3.56	0.63	3.49	0.59
The second positive emotions	3.60	0.57	3.66	0.45	3.51	0.79	3.14	0.65
Learning motivation	4.88	1.00	4.29	1.13	4.16	1.35	3.89	1.12
ICL	3.32	2.40	4.23	2.12	4.65	2.39	4.44	2.44
ECL	1.79	1.73	2.87	1.71	2.15	1.63	2.12	1.79
GCL	7.54	1.12	6.50	2.06	7.14	2.02	6.60	2.08
Retention test	13.55	5.39	12.96	5.84	10.76	5.23	13.56	5.44
Transfer test	3.21	1.91	3.12	1.67	2.48	1.67	3.21	1.65

*Affective PA, affective pedagogical agent; Neutral PA, neutral pedagogical agent; CR strategy, cognitive reappraisal strategy; ES strategy, expressive suppression strategy; ICL, intrinsic cognitive load; ECL, external cognitive load; GCL, germane cognitive load.*

#### Were Affective Pedagogical Agents Effective in Arousing Learners’ Positive Emotions?

**FIGURE 3:**
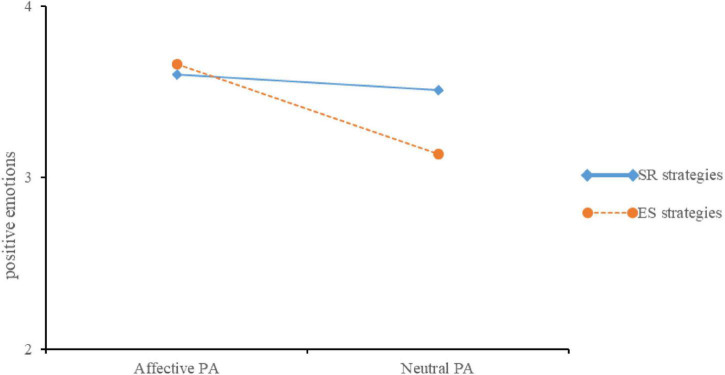
The positive emotions on the second measurement point for the four groups in Experiment 2.

For the second positive emotions measurement, there was a significant main effect of affective PA, *F*(1,106) = 5.10, *p* = 0.026, $\eta_{\text{p}}^{2}$ = 0.046, with the affective PA group reported more positive emotions (*M* = 3.63, *SD* = 0.47) than the neutral PA group (*M* = 3.34, *SD* = 0.75). There was no main effect for emotion regulation strategies, *F*(1,106) = 1.78, *p* > 0.05. The interaction between affective PA and emotion regulation strategies was significant, *F*(1,106) = 4.16, *p* = 0.044, $\eta_{\text{p}}^{2}$ = 0.04. The simple effects analysis found that there was a significant difference between conditions for learners who used ES strategy, *F*(1,106) = 8.89, *p* < 0.001, *d* = 0.99 (see Figure 3), with participants in the affective PA condition (*M* = 3.66, *SD* = 0.35) reported more positive emotions than those in the neutral PA condition (*M* = 3.14, *SD* = 0.65). Whereas there was no significant difference between two conditions for learners who used CR strategy, *F* < 1.

#### Were Affective Pedagogical Agents Effective in Improving Learners’ Intrinsic Motivation?

For the intrinsic motivation, the two-way ANCOVA identified a significant main effect of affective PA, *F*(1,106) = 4.02, *p* = 0.047, $\eta_{\text{p}}^{2}$ = 0.04. Students in the affective PA group (*M* = 4.60, *SD* = 1.09) reported higher level of intrinsic motivation than those in the neutral PA group (*M* = 4.03, *SD* = 1.25). The main effect of emotion regulation strategies was also significant, *F*(1,106) = 4.35, *p* = 0.04, $\eta_{\text{p}}^{2}$ = 0.04, with learners who used CR strategy (*M* = 4.50, *SD* = 1.24) had higher level of intrinsic motivation than learners who use ES strategy (*M* = 4.08, *SD* = 1.13). The interaction between these two factors was not significant, *F* < 1.

#### Were Affective Pedagogical Agents Effective in Facilitating Cognitive Processing and Learning Outcomes?

Concerning the ICL, the two-way ANCOVA revealed that there was no significant main effect of affective PA, *F*(1,106) = 1.21, *p* > 0.05, no significant main effect of emotion regulation strategies, *F* < 1, and no significant interaction between affective PA and emotion regulation strategies, *F* < 1. Concerning the ECL, the two-way ANCOVA revealed that there was no significant main effect of affective PA, *F*(1,106) = 1.42, *p* > 0.05, no significant main effect of emotion regulation strategies, *F*(1,106) = 2.96, *p* > 0.05, and no significant interaction between affective PA and emotion regulation strategies, *F*(1,106) = 1.74, *p* > 0.05. Concerning the GCL, the two-way ANCOVA revealed that there was a significant main effect of emotion regulation strategies, *F*(1,106) = 4.94, *p* = 0.028, $\eta_{\text{p}}^{2}$ = 0.05. Learners who use CR strategy (*M* = 7.33, *SD* = 1.65) reported higher GCL than learners who used ES strategy (*M* = 6.55, *SD* = 2.05). However, there was no main effect of affective PA, *F* < 1, and interaction between affective PA and emotion regulation strategies, *F* < 1.

For the retention test, the two-way ANCOVA revealed that there was no significant main effect of affective PA, *F* < 1, no significant main effect of emotion regulation strategies, *F*(1,106) = 1.83, *p* > 0.05, and no significant interaction between affective PA and emotion regulation strategies, *F* < 1. For the transfer test, the two-way ANCOVA revealed that there was no significant main effect of affective PA, *F* < 1, no significant main effect of emotion regulation strategies, *F*(1,106) = 1.10, *p* > 0.05, and no significant interaction between affective PA and emotion regulation strategies, *F* < 1.

### Discussion

These results of Experiment 2 replicate the findings of Experiment 1 and are partly consistent with hypothesis 1, indicating that there was a consistent pattern in which the affective PA group reported more positive emotions and higher level of motivation, but not performed better than the neutral PA group. In addition, consistent with hypothesis 2, affective PAs evoked positive emotions in learners who are accustomed to using expressive suppression strategy, but the positive effect disappeared for learners who used cognitive reappraisal strategy. Cognitive reappraisal strategy is related to expressing more positive emotions and less negative emotions, learners who used cognitive reappraisal strategy have confidence in managing and regulating their emotions to maintain positive emotional experience (Goldin et al., 2008). Expressive suppression is related to expressing more negative emotions and less positive emotions, learners who use expressive suppression strategy are more likely to feel intensity of negative emotions (Dryman and Heimberg, 2018). In comparison with learners who use cognitive reappraisal strategy, learners who use expressive suppression strategy have difficulty in regulating their emotions during learning. Therefore, direct instructional design such as adding an affective PA to the computer screen could provide affective support and help them to up-regulate positive emotions. Contrary to hypothesis 2, affective PAs improved intrinsic motivation of both learners who used cognitive reappraisal strategy and those who used expressive suppression strategy. The reason may be that learners under the affective PA condition experienced more positive emotions, while positive emotions can enhance learning motivation and interest (Um et al., 2012). Therefore, learners who used either emotion regulation strategy reported higher motivation in affective PA condition. In addition, affective PAs did not help improve the cognitive processing and learning outcomes of learners who used expressive suppression strategy. In the present study, affective PAs were more effective for learners’ affective processing (e.g., emotions and motivation) but not for cognitive processing. The small effects of affective PAs on cognitive activities may not affect the cognitive outcomes of learners who use expressive suppression strategy.

In conclusion, Experiment 2 identified that the moderating effect of learners’ emotion regulation strategies in the effectiveness of affective PAs. To clarify, affective PAs can better exert their positive influence on positive emotions when learners used expressive suppression strategy. The result showed that adopting cognitive reappraisal strategy may help learners to experience more positive emotions by changing the negative cognitions to regulate emotions experienced in learning.

## Experiment 3

The results of Experiment 2 replicated the findings of Experiment 1 that affective PAs could increase learners’ positive emotions and motivation. Moreover, Experiment 2 found the moderating effect of learners’ emotion regulation strategies on positive emotions. In addition to the emotion regulation strategies learners used, learners’ prior knowledge also plays an important role in learning. In Experiment 3, we aim at further exploring whether the effects of affective PAs depend on learners’ prior knowledge. Specifically, whether affective PAs are more helpful to students with low prior knowledge than those with high prior knowledge.

### Method

#### Participants and Design

Three hundred and eighteen undergraduates were recruited to complete a prior knowledge test about synaptic transmission. Then, according to their scores on prior knowledge (*M* = 12.06, *SD* = 7.09), the top 27% and the bottom 27% of the students were, respectively, classified as high prior knowledge (HPK) group and low prior knowledge (LPK) group. The final sample consisted of 102 participants. A power analysis with G*Power 3.1 was conducted to calculate the number of participants with a medium effect size of f = 0.30 with power set at 0.80 and alpha set to 0.05 (Erdfelder et al., 2009). The recommended sample size was 102 participants. Among them, 52 were high knowledge learners and 50 were low knowledge learners. An independent sample *t*-test showed that the prior knowledge score of high knowledge learners was significantly higher than low knowledge learners, *t*(100) = 32.99, *p* < 0.001, *d* = 6.54. The average age of the participants was 19.75 years (*SD* = 1.21), and 80 of them were women.

The experiment used a 2 × 2 between-subjects design with affective PA (affective PA vs. neutral PA) and prior knowledge (HPK vs. LPK) as factors. The participants were randomly assigned to four groups: 25 in the affective PA/HPK; 27 in the neutral PA/HPK; 25 in the affective PA/LPK; 25 in the neutral PA/LPK. There were no significant differences among four group on positive emotions, *F*(3,98) = 1.06, *p* > 0.05, and proportion of men and women, χ^2^(3) = 0.64, *p* > 0.05.

#### Materials and Apparatus

The learning materials, pretest (prior knowledge test and emotional state scale) and posttest (motivation questionnaire, cognitive load scale, retention test and transfer test) were the same as that in Experiment 1. Inter-rater reliability was *r* = 0.99 (*p* < 0.001) for the retention test and *r* = 0.96 (*p* < 0.001) for the transfer test. The apparatus was the same as Experiment 1.

#### Procedure

The procedure was the same as in Experiment 1. In addition, participants with high/low prior knowledge were randomly assigned to two groups.

### Results

Table 3 shows the means and standard deviations of each group for all variables. To explore the effects of affective PA and prior knowledge on learners’ positive emotions, intrinsic motivation, cognitive load and learning outcomes, we conducted a two-way ANOVA with affective PA (affective PA vs. neutral PA) and prior knowledge (HPK vs. LPK) as factors.

**TABLE 3:** Means and standard deviations of all tests for four groups in Experiment 3.

Dependent variables	Affective PA				Neutral PA			
	HPK		LPK		HPK		LPK	
	*M*	*SD*	*M*	*SD*	*M*	*SD*	*M*	*SD*
Prior knowledge	6.04	4.11	5.10	3.46	5.22	3.54	4.52	2.81
The first positive emotions	3.52	0.57	3.29	0.56	3.50	0.45	3.36	0.54
The second positive emotions	3.72	0.52	3.48	0.47	3.46	0.52	3.03	0.72
Learning motivation	4.93	0.81	4.49	0.69	4.61	0.93	3.78	1.61
ICL	2.72	2.18	5.67	1.84	3.05	1.94	6.20	1.77
ECL	2.03	1.19	3.37	1.96	2.16	1.36	3.07	1.37
GCL	7.46	1.14	7.14	1.07	6.68	1.88	6.59	1.14
Retention test	16.88	2.19	8.55	4.14	15.65	2.27	8.74	3.54
Transfer test	3.85	1.52	1.72	0.85	4.19	1.36	2.01	1.82

*Affective PA, affective pedagogical agent; Neutral PA, neutral pedagogical agent; HPK, high knowledge learners; LPK, low knowledge learners; ICL, intrinsic cognitive load; ECL, external cognitive load; GCL, germane cognitive load.*

#### Were Affective Pedagogical Agents Effective in Arousing Learners’ Positive Emotions?

For the second positive emotions measurement, the analysis revealed a significant main effect for the affective PA, *F*(3,98) = 9.91, *p* = 0.002, $\eta_{\text{p}}^{2}$ = 0.09. Learners in the affective PA group reported more positive emotions (*M* = 3.69, *SD* = 0.50) than learners in the neutral PA group (*M* = 3.25, *SD* = 0.65). The main effect of prior knowledge was also significant, *F*(1,98) = 8.79, *p* = 0.004, $\eta_{\text{p}}^{2}$ = 0.08. Learners with high prior knowledge (*M* = 3.58, *SD* = 0.53) had more positive emotions than learners with low prior knowledge (M = 3.25, *SD* = 0.64). However, there was no interaction effect for the two factors, *F*(1,98) < 1, *p* > 0.05.

#### Were Affective Pedagogical Agents Effective in Improving Learners’ Intrinsic Motivation?

The two-way ANOVA computed on intrinsic motivation scores revealed a main effect of affective PA, *F*(1,98) = 7.84, *p* = 0.006, $\eta_{\text{p}}^{2}$ = 0.07. Learners in the affective PA group (*M* = 4.71, *SD* = 0.77) reported higher level of intrinsic motivation than learners in the neutral PA group (*M* = 4.21, *SD* = 1.14). The main effect of prior knowledge was also significant, *F*(1,98) = 11.98, *p* = 0.001, $\eta_{\text{p}}^{2}$ = 0.11. Learners with high prior knowledge (*M* = 4.76, *SD* = 0.88) had higher level of intrinsic motivation than learners with low prior knowledge (*M* = 4.13, *SD* = 1.04). However, there was no interaction effect for the two factors, *F*(1,98) = 1.08, *p* > 0.05.

#### Were Affective Pedagogical Agents Effective in Facilitating Cognitive Processing and Learning Outcomes?

With regard to ICL, the two-way ANOVA revealed a main effect of prior knowledge, *F*(1,98) = 63.07, *p* < 0.001, $\eta_{\text{p}}^{2}$ = 0.39. Learners with high prior knowledge reported less ICL (*M* = 2.89, *SD* = 2.05) than learners with low prior knowledge (*M* = 5.94, *SD* = 1.87). There were neither a main effect of affective PA, *F*(1,98) = 1.24, *p* > 0.05, nor an interaction effect between these two factors, *F* < 1. For the ECL, the two-way ANOVA revealed a main effect of prior knowledge, *F*(1,98) = 14.47, *p* < 0.001, $\eta_{\text{p}}^{2}$ = 0.13. Learners with high prior knowledge reported less ECL (*M* = 2.10, *SD* = 1.27) than learners with low prior knowledge (*M* = 3.22, *SD* = 1.68). There were neither a main effect of affective PA, *F* < 1, nor an interaction effect between these two factors, *F* < 1. For the GCL, the two-way ANOVA revealed a main effect of affective PA, *F*(1,98) = 4.26, *p* = 0.042, $\eta_{\text{p}}^{2}$ = 0.04. Learners in the affective PA reported more GCL (*M* = 7.30, *SD* = 1.10) than learners in the neutral PA group (*M* = 6.64, *SD* = 1.99). There were neither a main effect of prior knowledge, *F* < 1, nor an interaction effect between these two factors, *F* < 1.

For the retention test, there was a significant main effect of prior knowledge, *F*(1,98) = 139.63, *p* < 0.001, $\eta_{\text{p}}^{2}$ = 0.59. Learners with high prior knowledge (*M* = 16.24, *SD* = 2.19) performed better than learners with low prior knowledge (*M* = 8.55, *SD* = 4.14). There were neither a significant effect of affective PA, *F* < 1, nor an interaction between affective PA and learners’ prior knowledge, *F*(1,98) = 1.52, *p* > 0.05. For the transfer test, there was a significant main effect of prior knowledge, *F*(1,98) = 57.64, *p* < 0.001, $\eta_{\text{p}}^{2}$ = 0.37. Learners with high prior knowledge (*M* = 4.02, *SD* = 1.43) performed better than learners with low prior knowledge (*M* = 1.87, *SD* = 1.42). There were neither a significant effect of affective PA, *F*(1,98) = 1.22, *p* > 0.05, nor an interaction between affective PA and learners’ prior knowledge, *F* < 1.

### Discussion

Overall, as in Experiments 1 and 2, the results of Experiment 3 supported the idea that affective PAs could increase learners’ positive emotions and intrinsic motivation, but did not result in higher retention and transfer test scores. The results are partly consistent with hypothesis 1. However, the results are not consistent with hypothesis 3 that affective PAs would be more beneficial for low knowledge learners but not for high knowledge learners because this pattern was not found for positive emotions, intrinsic motivation and learning outcomes. One possible reason may be that the emotional state of one person is automatically affected by another person’s emotional expression (Hatfield et al., 1994, 2014), therefore, when provided with an affective PA, learners tend to mimic the emotions of instructor and synchronize their emotions with instructors’ facial expressions and voices irrespective of their prior knowledge. The learning results can be explained by considering cognitive load. Although both low prior knowledge learners and high prior knowledge learners invested high GCL to comprehend information, high prior knowledge learners experienced lower ICL and ECL than low prior knowledge learners. Therefore, high prior knowledge learners may perceive the learning material easier, and thus performed better than low prior knowledge learners in both PA conditions. In addition, the range of prior knowledge may affect the interaction effects between affective PAs and learners’ prior knowledge. More specifically, most of the participants were not complete novices or experts, thereby the levels of prior knowledge in synaptic transmission between the low and high group were not enough to span the entire continuum (Spanjers et al., 2011; Wang F. et al., 2020). In such cases, low prior knowledge learners may store some relevant knowledge structures in long-term memory, which might lead to that affective PA was not more beneficial to students with low prior knowledge.

In conclusion, Experiment 3 revealed that learners’ prior knowledge did not moderate the effectiveness of affective PAs and identified a strong experience dominance effect that learners with high prior knowledge performed better than those with low prior knowledge.

## General Discussion

### Empirical Contributions

In the present study, three experiments were conducted to investigate the effects of affective PAs on learners’ positive emotions, intrinsic motivation, and learning outcomes. Across three experiments, students reported more positive emotions and higher level of intrinsic motivation when adding an affective PA to the multimedia lesson on synaptic transmission. The effective sizes for positive emotions and motivation were strong and consistent: *d*_positive emotions_ = 0.70, 0.46, and 0.60 in Experiment 1, 2, and 3; *d*_intrinsic motivation_ = 0.76, 0.49, and 0.51 in Experiment 1, 2, and 3. This is the major empirical contribution of this research, which provides powerful evidence for the effects of adding an affective PA to an online lesson.

In addition, in Experiment 2, the affective PAs were more beneficial for learners who used expressive suppression strategy (*d*_positive emotions_ = 0.99) but not for learners who used cognitive reappraisal strategy (*d*_positive emotions_ = 0.13). However, in Experiment 3, affective PAs aroused positive emotions and intrinsic motivation of both high knowledge learners and low knowledge learners. This is another primary contribution of this study, indicating that emotion regulation strategies but not prior knowledge was a boundary condition for the effectiveness of affective PAs.

### Theoretical Implications

The pattern of results partially supports the emotional response theory (Horan et al., 2012) and the cognitive affective theory of learning with media (CATLM, Moreno and Mayer, 2007), which believes that students who study with affective PAs will have more positive emotions, higher level of intrinsic motivation and learn better than those who learn with neutral PAs. Our results indicated that affective PAs with smiling faces and enthusiastic voices could affect learners’ emotional and motivational states, but not learning performance.

Furthermore, the present study found that the beneficial effects of affective PAs on positive emotions were obtained for students who used expressive suppression strategy but not learners who used cognitive reappraisal strategy, which provided reliable empirical evidence for the individual difference assumption of the CATLM and expanded prior studies by identifying the important role of learners’ characteristics in understanding the effects of affective PAs.

### Practical Implications

Recent advances in computer technology have highlighted the important role of remote learning, online instruction, and learning with videos. In such cases, it is important for instructional practitioners to design video lessons as efficacious as possible. In the present study, we found that learners experienced more positive emotions and had a higher level of intrinsic motivation. Therefore, an important practical implication is that instructional designers should consider adding an affective PA who exhibited smiling facial expressions and enthusiastic voices when designing video lectures. In addition, it is important for instructors to display happy emotions either in traditional classrooms or in online courses to help increase students’ positive emotions and motivation.

In addition, this study showed that affective PAs were partially helpful in arousing positive emotions in learners who used expressive suppression strategy but not those who used cognitive reappraisal strategy. Therefore, another practical consideration is that instructional designers should take the characteristics of learners into account when adding an affective PA to the computer screen.

### Limitations and Future Directions

Notwithstanding these findings, this research has several limitations that should be addressed. First, participants were given the posttest immediately after the lesson. However, there are great differences in the learning outcomes of an immediate test and a delay test. The effects of affective PAs may be more pronounced in a delayed test (Horovitz and Mayer, 2021). Future research should add a delay test to explore the effects of affective PAs on learning.

Secondly, in this study, smiling facial expressions and enthusiastic voices were used to design the affective PAs. However, the current study did not distinguish the role of different emotional cues. Thus, an important issue for future research is to explore which emotional cues is most effective in learners’ emotions, motivation, and learning.

Thirdly, this study focused on undergraduate students as participants. In addition, all of the three experiments used the same learning materials concerning synaptic transmission and animation duration was short. Accordingly, whether these findings can be generalized to different disciplines, groups and longer learning time remain to be further explored. What needs special attention is that learners’ emotions are related to the perceived materials difficulty (Efklides and Petkaki, 2005). Therefore, it is also an interesting issue to examine whether the types and difficulty of learning materials moderate the effectiveness of affective PAs.

Finally, this study only used self-reported measures to explore the cognitive processing during learning. Further studies should use direct measurement techniques (e.g., eye tracking) to examine whether affective PAs serve as distractors or complements, thus elucidating the underlying mechanisms in learning with affective PAs.

## Conclusion

The present study demonstrated the benefits of adding an affective PA to a multimedia lesson, as indicated by more positive emotions and higher level of intrinsic motivation. In addition, this study examined the boundary conditions of affective PA effects, and found that affective PAs could arouse positive emotions in learners who use expressive suppression strategy but not in those who use cognitive reappraisal strategy. However, learners’ prior knowledge did not moderate the effects of affective PAs. These findings provide new perspectives for empirical research in the field of affective PAs, and also have important implications for educational practice.

## Data Availability Statement

The raw data supporting the conclusions of this article will be made available by the authors, without undue reservation.

## Ethics Statement

The studies involving human participants were reviewed and approved by the Ethical Committee of the School of Psychology at Central China Normal University. The patients/participants provided their written informed consent to participate in this study.

## Author Contributions

YQW contributed to the writing – original draft, data curation, visualization, interpretation, and methodology. XF contributed to the conceptualization, methodology, and data collecting. JG contributed to the analysis and writing. SG contributed to the conceptualization, interpretation, revising the work, supervision, funding acquisition, and validation. YNW and JW revised the draft. All authors have read and agreed to the published version of the manuscript.

## Conflict of Interest

The authors declare that the research was conducted in the absence of any commercial or financial relationships that could be construed as a potential conflict of interest.

## Publisher’s Note

All claims expressed in this article are solely those of the authors and do not necessarily represent those of their affiliated organizations, or those of the publisher, the editors and the reviewers. Any product that may be evaluated in this article, or claim that may be made by its manufacturer, is not guaranteed or endorsed by the publisher.
